# Research on prostate brachytherapy puncture control strategy based on adaptive PID control with FBG sensors

**DOI:** 10.1371/journal.pone.0329065

**Published:** 2025-08-13

**Authors:** Jianqiao Li, Xuesong Dai, Peng Li

**Affiliations:** 1 Faculty of Engineering, Monash University, Melbourne, Australian; 2 Automation College, Wuxi University, Wuxi, China,; 3 Mechanical Engineering, Tianjin University, Tianjin, China; 4 School of Automation, Nanjing University of Information Science and Technology, Nanjing, China; UN Mehta Institute of Cardiology and Research Center, INDIA

## Abstract

This paper enhances prostate brachytherapy robot accuracy by developing a needle deflection prediction model and a controlled puncturing strategy, addressing current challenges and trends. The study addresses the challenges in needle deflection prediction by proposing a correction force-based prediction model. The puncture control strategy comprises two phases: preoperative needle trajectory planning and intraoperative approach adjustment, both relying on corrective force. During operative adjustment, a model predicting and counteracting needle tip deflection ensures accurate corrective force application. An adaptive PID controller, utilizing Reinforcement Learning (RL), regulates corrective force for precise puncture accuracy. A dedicated experimental platform was constructed to validate the puncture control strategy for prostate seed implantation. The seed implantation’s average error was 1.96 mm, with a standard error of 0.56 mm. Experiments show that correction force in the strategy significantly reduces tip deflection, enhancing seed implantation precision.

## Introduction

Among the incidence of malignant cancers in men, prostate cancer has risen to the second highest place and the fifth leading cause of cancer death in men [1]. At present, the treatment of prostate cancer is mainly radical resection, external radiation therapy (EBRT) and Low dose rate (LDR) prostate brachytherapy (BT), supplemented by other treatment methods to achieve the best surgical effect [2]. Compared with radical resection, prostate cancer particle implantation has the characteristics of less trauma, faster recovery, fewer complications, and low hospital costs, and Ennis [3] concluded through a large number of clinical studies that BT can achieve similar treatment effects as radical resection, and has become the most desired treatment for patients. BT involves placing radiation sources inside or near the targeted treatment area. By utilizing the continuous radiation emitted by the radioactive particles, the structure and activity of tumor cells are affected, thereby selectively eliminating the tumor cells. Compared to traditional surgeries such as radical resection, radioactive particle implantation treatment has lower risks of side effects and offers better prognosis and quality of life. Currently, radioactive particle implantation therapy has become the standard treatment for early-stage prostate cancer in the United States [4].

**Fig 1:**
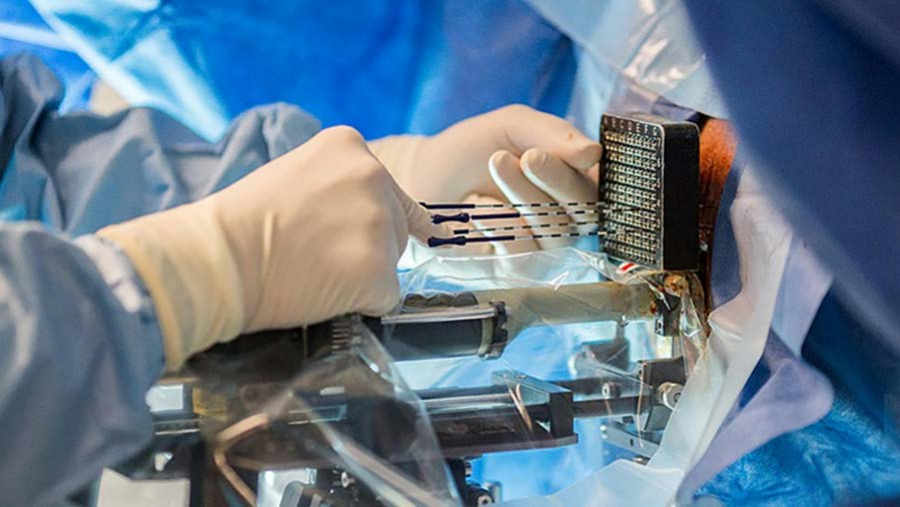
Current clinical treatment methods of brachytherapy.

In clinical practice, BT is primarily performed by doctors manually using a percutaneous puncture technique. As shown in Fig 1, a puncture needle is guided along a planned path to implant radioactive particles such as Iodine-125 and Palladium-103 into the tumor target area. Multiple small radiation sources emit continuous, short-range radiation to irradiate the tumor tissue. During the brachytherapy process, the dose distribution requirements for the tumor target area are quantitative and non-uniform, depending on the differences in the location of the tumor lesion in each patient. The position of each radioactive particle is adjusted to meet the radiation dose requirements of the tumor target area.

However, due to the steep radiation dose gradient [5–7], there are high precision demands for the placement of radioactive particles. Currently, due to limitations such as insufficient manual operation accuracy, unexpected organ movements, and physiological structures such as bones and blood vessels, it is often difficult in clinical settings to accurately place the radioactive particles in the predetermined position. This can result in incomplete coverage of the tumor target area, increasing the risk of tumor recurrence. Therefore, achieving precise implantation of radioactive particles into the target site has become a critical challenge that needs to be addressed in particle implantation therapy.

In clinical practice, doctors intermittently rotate the needle to control its linear progression. They rotate the needle to alter the direction of the needle tip’s bevel, enabling it to move in the opposite direction; however, manually controlling the precise path of the needle tip is challenging [4,5].Therefore, in recent years, robot-assisted BT technology has increasingly gained attention [6]. Research institutions achieve precise puncturing by guiding needle rotation, developing needle-tissue interaction models, creating needle deflection prediction models, and improving needle steering control. The basic interactions between needle and tissue, including stiffness force, friction, and cutting force, have been studied [7–10].Needle deflection prediction models include mechanical models [8–14] and kinematic models [15–20]. However, current kinematic models have little correlation with the characteristics of the punctured tissue, leading to discrepancies between the model and the actual trajectory. Mechanics-based needle deflection models take into account tissue properties and have led to improved needle deflection prediction models [10,11,13,14,17,21], providing information for axial needle rotation steering in model-based controllers [8,14–22]. In clinical practice, doctors use two methods to adjust the needle tip position during surgery: 1) rotating the needle body; 2) applying corrective force near the insertion point [22].Rotating the needle body is a simple operation, but it can cause adhesion between the patient’s tissue and the needle, leading to secondary injury to the patient. Method 2 requires the doctor to apply a corrective force perpendicular to the direction of needle insertion to steer the needle. However, the precision in the magnitude and timing of this force demands high skill from the doctor; improper application can lead to tearing of patient’s tissue. In recent years, the application of robotic technology in puncture procedures has become one of the hot topics in medical robotics research. Lehmann conducted puncture experiments on silicone tissue using a robot, studying the impact of corrective force on the precision of the puncture [23–25]. During the puncture process, the corrective force is applied directly to the needle body along the direction of needle deflection to reduce the deflection value. The advantage of this method is that the corrective force provides a continuous control input. The PID (Proportional, Integral, and Derivative) control used in the Ref. [25] is based on the calculation of proportional, derivative, and integral components. It exhibits a certain degree of lag, affecting operational efficiency. Research on the second method, the corrective force guidance technique, is currently in its initial stages. The control models based on this method need improvements in terms of accuracy and real-time performance.

## Materials and methods

### Needle deflection prediction model

**Fig 2:**
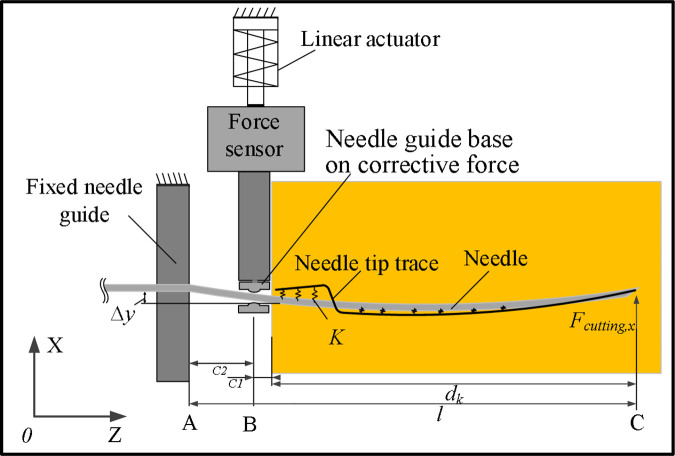
Schematic diagram of corrective force affecting needle deflection.

As shown in Fig 2, the left side of the puncture needle is fixed by the fixed needle guide, so only the needle shaft part from point A to point C is considered for modeling, which simplifies the model complexity and improves the computational efficiency of the mathematical model. During needle puncture, as the depth of puncture increases, the length of the needle from point A to point C is also increasing, so the length of the needle is a variable. At points B and C, the needle is subject to correction force and cutting force, respectively. The needle deflection prediction model is established by using the principle of minimum potential energy. Equations translate the functional work performed on the needle-tis sue system by energy and outside forces stored in the needle and the tissue during puncture into a linear equation system by applying the Rayleigh-Ritz approach [26]. Finally, by using the principle of minimum potential energy to solved the linear equations of the needle deflection.

The system energy ∏(u) for needle-tissue is expressed as:


$$\begin{array}{c} \prod (u)=U(u)+V\\ =U_{s}(u)+U_{d}(u)+V_{l}+V_{t} \end{array}$$ (1)

Where: U(u) is the energy possessed by the system itself; V is the energy generated on the system by the lateral driving force and the cutting reaction force; $U_{s}(u)$ is the elastic potential energy generated by the deflection of the needle; $U_{d}(u)$ is the compression potential energy generated when the needle is inserted into the tissue and the tissue is compressed; $V_{l}$ is the energy generated by the work done by the corrective force $F_{l}$; $V_{t}$ is the energy generated by the work done by the component *F*_*cutting,x*_ of the X-axis cutting force.

(1) Elastic potential energy of needle $U_{s}(u)$

In this paper, the axial deflection of the needle can be ignored, and only the radial deflection of the needle is considered. Elastic potential energy generated by needle deflection $U_{s}(u)$ can be expressed as:


$$U_{s}(u)=\int_{0}^{l}\frac{EI}{2}(\frac{\partial^{2}u{\left(z\right)}^{2}}{\partial z^{2}})dz$$ (2)

Where: E is Young ‘s modulus of puncture needle; I is moment of inertia; l is length of puncture needle; u(z) is deflection model of needle; *z* is depth of puncture

(2) Tissue compression potential energy $U_{d}(u)$

When the needle puncture into the tissue, the needle is deflected and occupies the space of original tissue, the tissue around the needle will be squeezed by the needle, and the energy $U_{d}(u)$ in the compressed tissue is expressed as:


$$U_{d}(u)=\frac{K}{2}\int_{l-d_{k}}^{l}{\left(u(z)-u_{t}(z)\right)}^{2}dz$$ (3)

Where: $u_{t}(z)$ is measured needle tip path, the value of *z* ranges from 0 to *l*; *d*_*k*_ is the final puncture depth of the need*l*e and *z* is the depth of puncture.

**Fig 3:**
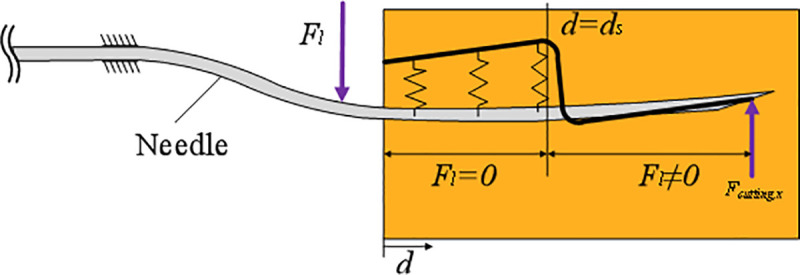
Schematic of needle deflection when corrective force is applied.

When the needle puncture into the tissue, the compressed tissue can be represented by virtual springs that join into a needle-shaped trajectory as shown in Fig 3. According to Eq 3, the elongation of elastic spring is related to the deviation position of needle shaft after receiving correction force and the difference between path $u_{t}(z)$ of needle tip.

(3) Work done by corrective force $V_{l}$

Apply a correction force perpendicular to the needle axis to point B of the needle axis by a corrective force application mechanism, and work done by the corrective force $V_{l}$ can be expressed as:


$$V_{l}=F_{l}u(c_{2})$$ (4)

Where: $u(c_{2})$ represents the offset distance of needle at point B.

(4) Work done by *F*_*cutting,x*_ (component of cutting force along X-axis)

The X-axis component force *F*_*cutting,x*_ of the cutting force is the main cause of needle deflection during needle puncture into tissue and is caused by the asymmetric geometry of the oblique needle tip. Because of the asymmetry of the needle tip, the tissue is squeezed by the needle tip as it passes through the tissue. Therefore, the needle will bend in the same direction as the bevel. Therefore, the direction of the bevel is responsible for determining both the sign of *F*_*cutting,x*_ and the direction in which the needle will deflect.

The work done by *F*_*cutting,x*_ is shown as follows:


$$V_{t}=F_{cutting,x}u(l)$$ (5)

Where: u(l) is the value of needle deflection

The meaning of u(l) is different from the meaning of $u_{t}(z)$. Needle tip path $u_{t}(z)$ is constituted by the tip deflection u(l) of the past puncture step and thus is dependent on the z-coordinate in the horizontal plane. In summary, the Eq 2–4 is substituted into Eq 1, and then the system energy ∏ can be expressed as:


$$\Pi (u)=\{\begin{array}{c} {}*20c\int_{o}^{l}\frac{EI}{2}(\frac{\partial^{2}u{\left(z\right)}^{2}}{\partial z^{2}})dz+\frac{K}{2}\int_{l-d_{k}}^{l}{\left(u(z)-u_{t}(z)\right)}^{2}dz-F_{cutting,\;x}u(d,l)\;(z<d_{l})\\ \int_{o}^{l}\frac{EI}{2}(\frac{\partial^{2}u{\left(z\right)}^{2}}{\partial z^{2}})dz+\frac{K}{2}\int_{l-d_{k}}^{l}\begin{array}{c} (u(z)-u_{t}(z){)}^{2}dz\\ -F_{l}u(c_{2})-F_{cutting,\;x}u(d,l)\;(z>d_{l}) \end{array} \end{array}$$ (6)

Where: *d*_*l*_ is the puncture depth when applying corrective force to needle.

In attempt to solve the energy-based needle-tissue system model that was presented before, the Rayleigh-Ritz approach was applied in order to find an answer to the problem of the needle’s deflection variable. According to the Rayleigh-Ritz method, an approximation of a differential equation that takes the form of a function can be found by adding a finite weighted shape function to itself. Function of weighting for series that are finite:


$$u_{n}(z)=\sum_{i=1}^{n}q_{i}(z)g_{i}$$ (7)

Where: $q_{i}(z)$ refers to the *i-th* shape function; $g_{i}$ refers to the weighting coefficient corresponding to the shape function. $q_{i}(z)$ can be calculated using the following equation [27]:


$$q_{i}(z)=\frac{1}{k_{i}}(\sin(\beta_{i}\frac{z}{l})-\sinh(\beta_{i}\frac{z}{l}))-\gamma_{i}[\cos(\beta_{i}\frac{z}{l})-\cosh(\beta_{i}\frac{z}{l})]$$ (8)

Where: $\gamma_{i}$ and $k_{i}$ can be calculated using the following formula:


$$\gamma_{i}=\frac{\sin\beta_{i}+\sinh\beta_{i}}{\cos\beta_{i}+\cosh\beta_{i}}$$ (9)

$$k_{i}=\sin\beta_{i}-\sinh\beta_{i}-\gamma_{i}(\cos\beta_{i}-\cosh\beta_{i})$$ (10)

Where: $\beta_{i}$ is the constant value in the cantilever model without clamping, when i > 4, $\beta_{1}=1.857$, $\beta_{2}=4.695$, $\beta_{3}=7.855$, $\beta_{4}=10.996$, $\beta_{i}\approx \pi (i-1/2)$.

Bringing Eq 8 into Eq 6, get the following formula:


$$\Pi (u_{n})=\frac{EI}{2}\int_{0}^{l}\left(\sum_{i=1}^{n}{q_{i}}^{\left(2\right)}(z)g_{i}\right)2dz+\frac{K}{2}{\left(\int_{l-d_{k}}^{l}\sum_{i=1}^{n}q_{i}(z)g_{i}-u_{t}(z)\right)}^{2}dz-F_{l}\sum_{i=1}^{n}q_{i}(c_{2})g_{i}-F_{t,x}\sum_{i=1}^{n}q_{i}(l)g_{i}\mathrm{\backslash ]}$$ (11)

Where: ${q_{i}}^{\left(2\right)}(z)$ represents the second derivative of $q_{i}(z)$ relative to *z*.

When $\partial \Pi /\partial g_{j}=0$, and the value range of *j* is (1,n), $\Pi (u_{n})$ gets the minimum value. Based on this condition, a system of linear equations with a weighted coefficient $g_{i}$ can be established and solved.

Then take the partial derivative of $g_{i}$ for $\Pi (u_{n})$, and it can be seen from the Eq 8 that for any i and j values, there is *q*_*i*_*(z)*=*q*_*j*_*(z)*=1, and for any *j* value, the value of *q*_*j*_*(c2)* can be found, so the following results can be obtained:


$$\frac{\partial \Pi (u_{n})}{\partial g_{j}}=EI\int_{0}^{l}(\sum_{i=1}^{n}q_{i}\prime \prime (z)g_{i})q_{j}\prime \prime (z)dz+K\int_{l-d_{k}}^{l}(\sum_{i=1}^{n}\begin{array}{c} q_{i}(z)g_{i}\\ -u_{t}(z) \end{array})q_{j}(z)dz-F_{l}q_{j}(z)-F_{cutting,x}=0$$ (12)

Simplifying Eq 12, extracting $g_{i}$ can get Eq 13, substituting and adding the values of *i* can get a simplified formula:


$$\sum_{i=1}^{n}\varphi_{ji}g_{i}-\omega_{j}-\gamma_{j}-F_{t,x}=0$$ (13)

Where:

$\varphi_{ji}(z)=EI\int_{0}^{l}q_{i}\prime \prime (z)q_{j}\prime \prime (z)dz+K\int_{l-d_{k}}^{l}q_{i}(z)q_{j}dz$; $\omega_{j}(z)=K\int_{l-d_{k}}^{l}u_{t}(z)q_{j}(z)dz$; $\gamma_{j}=F_{l}q_{j}(c_{2})$.

According to the above equations analysis, you can write a matrix formula with Eq 13:


$$\underset{\Phi }{\underbrace{\left[\begin{array}{ccc} {}*20c\varphi_{11} & \cdots & \varphi_{1n}\\ \vdots & \ddots & \vdots \\ \varphi_{n1} & \cdots & \varphi_{nm} \end{array}\right]}}=\underset{g}{\underbrace{\left[\begin{array}{c} {}*20cg_{l}\\ \vdots \\ g_{ln} \end{array}\right]}}=F_{l}\underset{q(c_{2})}{\underbrace{\left[\begin{array}{c} {}*20cq_{l}(c_{2})\\ \vdots \\ q_{n}(c_{2}) \end{array}\right]}}+F_{cutting,x}1_{n\times 1}+\underset{\Omega }{\underbrace{\left[\begin{array}{c} {}*20cw_{1}\\ \vdots \\ w_{n} \end{array}\right]}}$$ (14)

Where:$1_{n\times 1}$ represents a column vector of size *n*.

The unknown vector *g* can be solved according to Eq 14 as follows:

Where:$1_{n\times 1}$ represents a column vector of size n.

The unknown vector g can be solved according to Eq 14 as follows:


$$g=\Phi^{-1}(F_{l}q(c_{2})+F_{cutting,x}1_{n\times 1}+\Omega )$$ (15)

Substituting Eq 15 into Eq 7 calculates the deflection function $u_{n}(z)$ of the needle.

### Puncture control strategy based on corrective force

This chapter builds a preoperative puncture control strategy based on the needle flexure deformation prediction model established in Chapter 2, because there is a certain error between the needle flexure deformation prediction model and the actual system, and the puncture operation is easily interfered by external factors, resulting in deviation between the needle tip position and the expected position, in order to overcome the adverse effects of model uncertainty and external interference, while considering the complex model characteristics of the system, it is difficult to apply the robust control algorithm usually based on the model, and the ordinary proportional integral derivative (Proportional-Integral-Derivative, PID) controller has poor robust performance and is difficult to meet the system requirements of this paper, so this paper will build an adaptive PID (RL-APID) control system based on reinforcement learning (RL), which adjusts the corrective force in real time so that the needle tip can reach the target point.

### Preoperative needle trajectory planning

**Fig 4:**
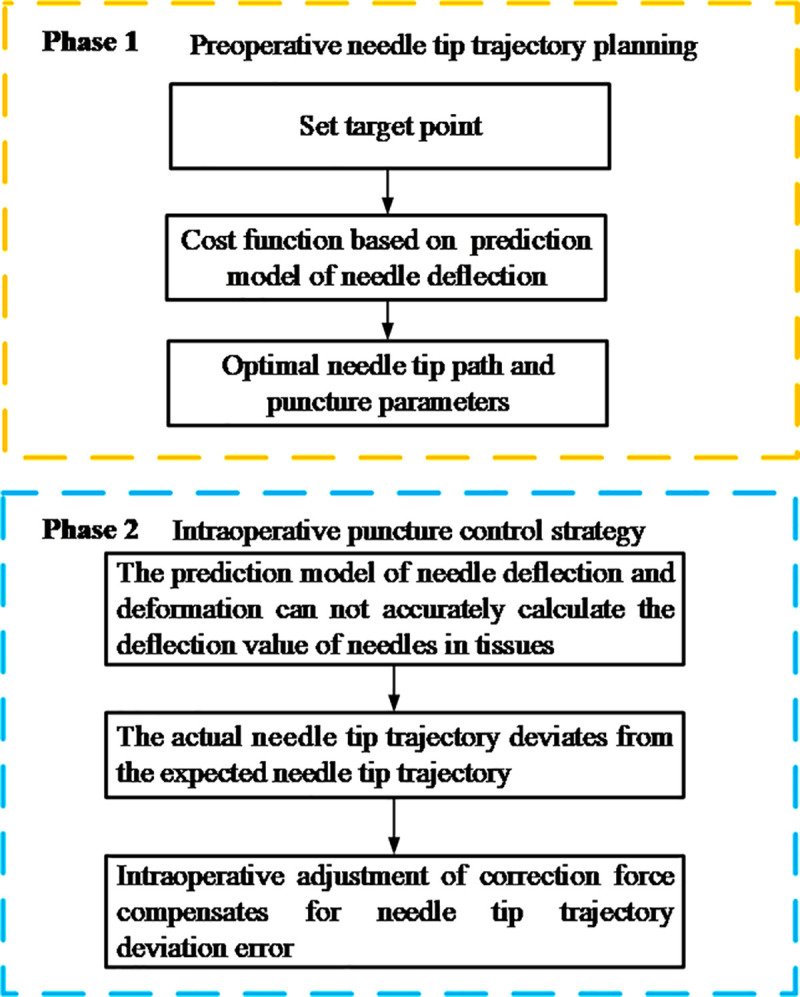
Schematic diagram of the overall puncture control strategy.

The puncture control strategy consists of two phases, as shown in Fig 4.

The phase 1 is the preoperative needle tip trajectory planning stage of the puncture needle. According to the needle deflection prediction model built in Chapter 2, the best needle tip path for the needle tip to reach the target point is obtained, and the corresponding puncture parameters-correction force *F*_*l*_ and puncture depth *d*_*k*_ are obtained.

**Fig 5:**
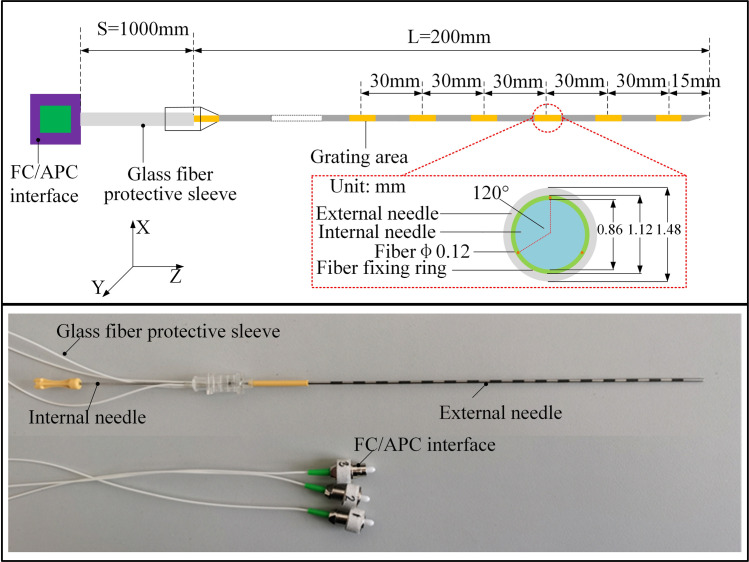
Structure diagram of FBG embedded needle.

The phase 2 is the intraoperative puncture control strategy stage of the needle. After applying the correction force *F*_*l*_, discrepancies arise between the intraoperative needle tip trajectory and the preoperative planned trajectory as the puncture needle is inserted. To accurately monitor and mitigate these errors, Fiber Bragg Grating (FBG) sensors are embedded within the needle(as shown in Fig 5), enabling precise sensing of the needle tip position in real-time. FBG sensors are mainly used to feedback forces, pressures and shapes, and the wavelength changes when the fibers elongate due to mechanical loads or changes in temperature. In this paper, the FBG sensor type is OSC1100−05. The main function of the FBG demodulator is to process the data collected by the FBG sensor in the corresponding software Enlight, which in turn converts it into the position information of the needle. Through the adaptive PID control strategy based on reinforcement learning, the size of the correction force is adjusted in real time to minimize the puncture error.

**Fig 6:**
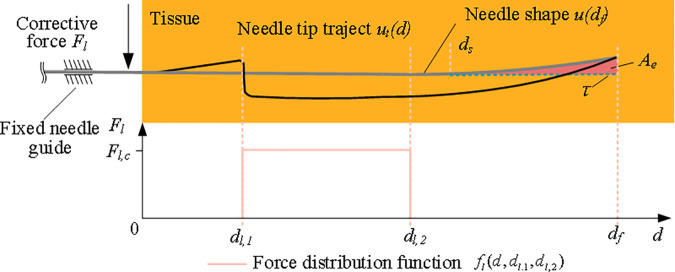
Schematic diagram of preoperative needle tip trajectory planning.

Before operation, first set the desired needle body line segment τ, As shown in Fig 6.

Based on the prediction model of needle deflection, the puncture parameters of the best needle tip trajectory were obtained. Based on the prediction model of needle deflection, the cost function to minimize *A*_*e*_ area is established. *A*_*e*_ is the area enclosed by the expected needle body line segment and the needle body line segment calculated by the model from *d*_*s*_ to *d*_*f*_. Considering that the search space of the correction force distribution function *f*_*l*_ is generally infinite, the simplified force distribution function *f*_*l*_ is selected to reduce the search space.


$$f_{l}(d,d_{l\mathrm{.1}},d_{l,2})=F_{l,c}[k(d-d_{l,1})-k(d-d_{l,2})]d\in (0,d_{f})$$ (16)

Where: k(·) is a step function.

*F*_*l,c*_, *d*_*l,1*_ and *d*_*l,2*_ indicate the magnitude of the corrective force, as well as the starting and ending depths at which the corrective force is applied. As shown in Eq 16, the corrective force distribution function $f_{l}$ is a function of *d*, *d*_*l,1*_,and *d*_*l,2*_. The cost function *R*(*F*_*l,c*_, *d*_*l,1*_) constructed by Eq 16 is the sum of squares of the residual between the desired value τ of the needle body segment and the shape of the final puncture depth.


$$R(F_{l,c},d_{l,1})=\sum_{z_{\tau }\in (d_{s},d_{f})}{\left(u(d_{f},z_{\tau },F_{l,c},d_{l,1})-\tau \right)}^{2}$$ (17)

where: $u(d_{f},z_{\tau },F_{l,c},d_{l,1})$ is the simulated deflection value of the needle at the final puncture depth obtained from the needle deflection prediction model.

**Fig 7:**
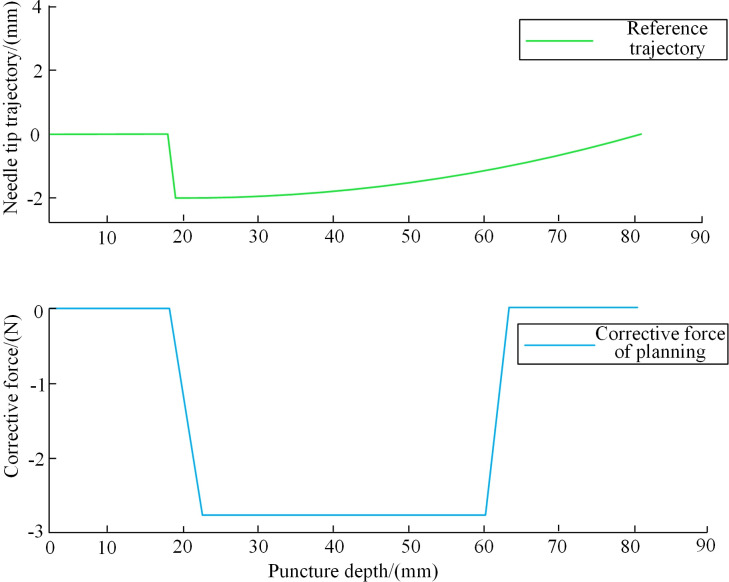
Optimal tip path and corresponding correction force.

The input of the cost function is the constant correction force *F*_*l,c*_ and the puncture depth *d*_*l,1*_.when *F*_*l,c*_ is applied that minimizes the value of *R*. Through the experiment, it is determined that the effective depth of the stop driven by the correction force *F*_*l,c*_ is 60 mm.The optimization algorithm is selected to find the optimal value of parameters. The optimization algorithm is selected to find the optimal value of parameters *F*_*l,c*_ and *d*_*l,1*_, so the optimal tip trajectory is the pattern search method. The *F*_*l,c*_ and *d*_*l,1*_ puncture parameters that make *R*(*F*_*l,c*_, *d*_*l,1*_) the minimum are obtained, and the identified optimal needle tip trajectory is used as the reference trajectory of intraoperative puncture control strategy during the puncture process. Simulate the preoperative puncture control strategy algorithm. The origin is the starting point, and the expected needle body segment is the segment with curvature of 0. Calculate the optimal path and the size of *F*_*l,c*_ and *d*_*l,1*_. The result is that a correction force of 2.8N is applied at 19 mm, as shown in Fig 7.

### Intraoperative puncture control strategy

1) Theoretical analysis of online adjustment

During puncture, the corrective force applied to the needle is adjusted according to the error between the pre-planned needle tip trajectory and the measured needle tip deflection value. In general, the corrective force predicted in the phase 1 (preoperative puncture strategy stage) can be used to control the puncture of the puncture needle. However, due to the errors in the needle deflection prediction model and the possible changes in conditions in the physical system, the prediction accuracy of the needle deflection prediction model cannot meet the requirements, so it is necessary to feed back the needle tip deflection value obtained from the FBG sensor and recalculate the corrective force online.

In order to predict the corrective force required to bring the needle tip from the current position to the target point, a reverse needle deflection prediction model based on the required needle deflection value is required to reverse the corrective force.

Reverse needle deflection prediction model:

$\delta_{e}=u_{e}(d+\Delta d)$ is the expected tip deflection value,

which is achieved by applying an undetermined corrective force *F*_l_*. Where, Δd is the feed distance of the puncture needle when the corrective force is applied. Assume that the trajectory of the needle tip *u*_*t*_*(d)* to the current depth d is known through measurement.

In order to solve the unknown correction force *F*_l_* to make the needle tip reach the ideal deflection value, first use vector $\Lambda ={\left[\begin{array}{cc} {}*20c0_{n\times 1} & \delta_{e} \end{array}\right]}^{T}$ to expand the dimension of Eq 14, move $F_{l}q(c_{2})$ to the right, and combine $q_{j}(c_{2})$ in $F_{l}q(c_{2})$ into Φ, we can get:


$$\underset{\Phi^{\Psi }}{\underbrace{\left[\begin{array}{cc} {}*20c\Phi & -q_{j}(c_{2})\\ {}[5pt]1_{n\times 1} & 0 \end{array}\right]}}=\underset{g\Psi }{\underbrace{\left[\begin{array}{c} {}*20cg\\ \vdots \\ F_{l}^{*} \end{array}\right]}}=\left[\begin{array}{c} {}*20cF_{cutting,x}1_{n\times 1}\\ {}[5pt]0 \end{array}\right]+\underset{\Omega^{\Psi }}{\underbrace{\left[\begin{array}{c} {}*20c\Omega \\ \vdots \\ 0 \end{array}\right]}}+\underset{\wedge }{\underbrace{\left[\begin{array}{c} {}*20c0_{n\times 1}\\ \vdots \\ \delta_{e} \end{array}\right]}}$$ (18)

The final Eq 18 can be written as:


$$g^{\Psi }=\Phi^{\Psi -1}(F_{cutting,x}1_{n\times 1}+\Omega^{\Psi }+\Lambda )$$ (19)

i.e., ${F_{l}}^{*}=g_{n+1}^{\Psi }$.

With the above Equations, given the parameters *K*, *F*_*cutting, x*_ and measuring needle tip track *u*_*t*_*(d)*, it is possible to predict the magnitude of the corrective force required to be the desired needle deflection value *δ*_*e*_. The advantage of this corrective force calculation method is that it does not require time-consuming iterative searches, which is key to the time constraint of a given sample during real-time trajectory replanning. During puncture, the corrective force is removed when the following criteria are met: 1) the maximum corrective force limit $F_{l,\max}$ is exceeded (the maximum value in this article is 4N);2) The limit of variation in corrective force between objects exceeds *d > d*_*l,2*_ (where the maximum *d*_*l,2*_ is 60 mm), and if any of these criteria are met, the reference force of the corrective force drive is set to 0. These conditions are all extreme cases that may exist when operating on the model. When extreme conditions occur, the reference force of the corrective force needle guide will be set to 0. The above is the modeling process of the reverse needle deflection deformation prediction model.

2) Intraoperative needle tip position adjustment based on reinforcement learning adaptive PID(RL-APID) control

This paper will design an adaptive PID controller based on reinforcement learning technology, adopt the reinforcement learning technology in the form of Actor Critical structure, and respectively use the radial basis function neural network (RBFNN) to realize the Actor and Critical mechanisms, which can effectively reduce the storage requirements and avoid repeated calculations, Then a new adaptive update rule of PID control is designed based on Actor Critic structure of RBFNN.

The main contributions of this paper are as follows: First, the one-step prediction output is considered, and the enhanced signal is redefined. Therefore, temporal difference (TD) includes prediction error; Secondly, the new adaptive update rule can be calculated according to TD error. Finally, the proposed scheme is modelless design, which is very suitable for complex practical systems that are difficult to obtain accurate mathematical models.

(1)Math problem description

In order to more clearly explain the design idea and process of RL-APID, first consider the following general form of discrete time nonlinear dynamic model


$$\begin{array}{c} x\left(t+1\right)=f\left(x\left(t\right)\right)+g\left(x\left(t\right)\right)u\left(t\right)\\ y\left(t\right)=h\left(x\left(t\right),u\left(t-1\right)\right) \end{array}$$ (20)

where: System state $x\left(t\right)\in R^{m}$ at time t, control input $u\left(t\right)\in R^{n}$, output y(t).

As the details of the allowable model are unknown in reinforcement learning technology, Eq 20 can be expressed in a more compact form as follows


$$\begin{array}{c} x\left(t+1\right)=F\left(x\left(t\right),u\left(t\right)\right)\\ y\left(t\right)=h\left(x\left(t\right),u\left(t-1\right)\right) \end{array}$$ (21)

In order to apply the reinforcement learning control technology to the Eq 21, the system first needs to meet the following two assumptions.

Assumptions 1: Because the state of Eq 21 at time t + 1 only depends on the state and input at time t, and has nothing to do with the historical state before time t and input information, Eq 21 satisfies the “memoryless” property of Markov chain. This assumption is defined in the framework of Markov Decision Process (MDP). The goal of MDP is to achieve specific goals through satisfactory control strategies. It is similar to the definition of reinforcement learning technology, so it has an important influence in the process of combining control problems with reinforcement learning technology.

Assumptions 2: The sign of partial derivatives of function h(·) with respect to all elements is known and the sign is the same as that of system Jacobian matrix. The sign of the partial derivative of a function with respect to all elements is known and is the same as the sign of the Jacobian matrix of the system.

Since the puncture closed-loop control system in this paper is easily affected by the jump of PID derivative term, this paper proposes a speed type PID control structure to reduce the adverse effects caused by the jump of derivative term. The discrete time control structure is designed as follows.


$$u\left(t\right)=u\left(t-1\right)+K_{I}\left(t\right)e\left(t\right)-K_{P}\left(t\right)\Delta y\left(t\right)-K_{D}\left(t\right)\Delta^{2}y\left(t\right)$$ (22)

From the Eq 22, the control increment is


$$\begin{array}{c} \Delta u\left(t\right)=K_{I}\left(t\right)e\left(t\right)-K_{P}\left(t\right)\Delta y\left(t\right)-K_{D}\left(t\right)\Delta^{2}y\left(t\right)\\ =K\left(t\right)\Theta \left(t\right) \end{array}$$ (23)

where: $K\left(t\right)=\left[K_{I}\left(t\right),K_{P}\left(t\right),K_{D}\left(t\right)\right]$ is the control parameter vector of the adaptive PID controller, define $\Theta \left(t\right)={\left[e\left(t\right),-\Delta y\left(t\right),-\Delta^{2}y\left(t\right)\right]}^{T}$ as the augmented system state, define $\Delta =1-z^{-1}$ is the difference operation symbol, which means the difference between the current time variable and the previous time variable. Therefore $\Delta^{2}y\left(t\right)$ can be further expanded and expressed as


$$\Delta^{2}y\left(t\right)=\Delta y\left(t\right)-\Delta y\left(t-1\right)=y\left(t\right)-2y\left(t-1\right)+y\left(t-2\right)$$ (24)

where: e(t) in Θ(t) is defined as the tracking error between the system reference input and the actual system output, that is, design e(t) is


$$e\left(t\right)=y_{d}\left(t\right)-y\left(t\right)$$ (25)

where: $y_{d}\left(t\right)$ is the reference input expected by the system.

**Fig 8:**
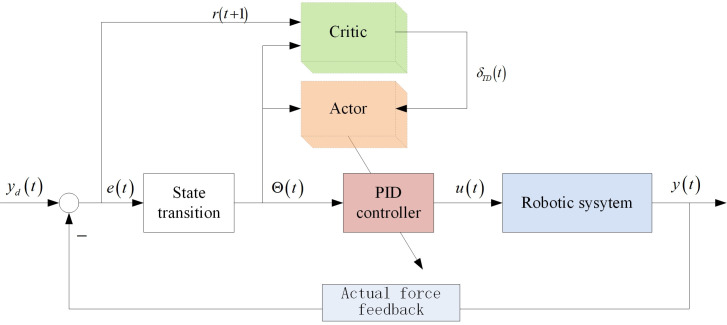
Structure block diagram of adaptive PID control method based on reinforcement learning.

The structure block diagram of the adaptive PID control method based on reinforcement learning proposed in this paper is shown in Fig 8. The input of the Actor Critical structure is Θ(t), which is converted from the trajectory tracking error e(t). The actuator Actor adjusts the controller online by using the observed system state, while the evaluator Critical not only receives the system state, but also receives the reward signal r(t+1), which evaluates the system performance and outputs the timing difference error.

Timing differential error $\delta_{TD}\left(t\right)$ is an important parameter in the design process. The purpose of this section is to design a PID control system with a new adaptive law using the Actor-Critic structure, while meeting the system tracking accuracy and robust performance requirements.

Adaptive control system design process:

First, define a value function in the following form


$$V\left(t\right)=\sum_{i=t}^{\infty }\gamma^{i-t}r\left(x\left(i\right),u\left(i\right)\right)$$ (26)

where: 0<γ≤1 is the attenuation factor, u(t) is the control signal, Function r(x(i),u(i)) is called a reward signal or reinforcement signal, it is generally designed as a quadratic function.

Rewrite Eq 26 as


$$V\left(t\right)=r\left(x\left(t\right),u\left(t\right)\right)+\gamma \sum_{i=t+1}^{\infty }\gamma^{i-\left(t+1\right)}r\left(x\left(i\right),u\left(i\right)\right)$$ (27)

Eq 27 is still an infinite summation equation and is difficult to solve, so it is further expressed as follows


V(t)=r(x(t),u(t))+γV(t+1),V(0)=0 (28)

Eq 28 is also known as the Bellman equation.

Based on Bellman’s Eq 28, the timing difference error can be defined as


$$\delta_{TD}\left(t\right)=r\left(x\left(t\right),u\left(t\right)\right)+\gamma V\left(t+1\right)-V\left(t\right)$$ (29)

If the Bellman equation holds, then the timing difference error $\delta_{TD}\left(t\right)=0$, so the control signal at each moment can be considered the optimal control strategy.

**Fig 9:**
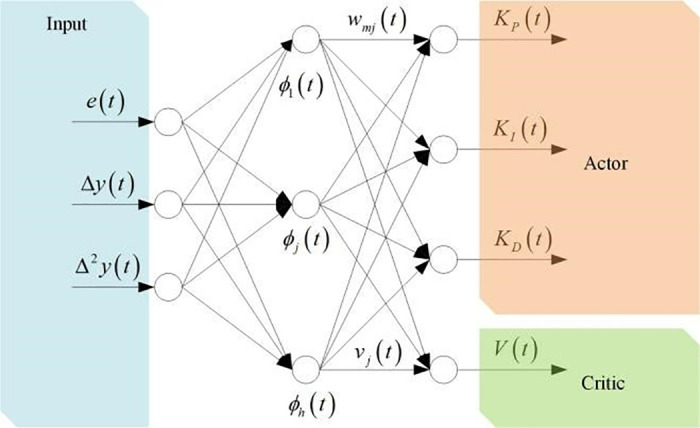
Block diagram of actor-critic structure.

RBF neural networks are widely used in parameter recognition due to their versatile approximation ability. In this paper, we will use the RBF neural network to implement the Actor-Critic structure, and the block diagram is shown in Fig 9.

The neural network structure consists of three layers of neuron nodes: input layer, hidden layer and output layer. The input layer is composed of trajectory tracking error and system output, RBF neural network transmits the system state from the input layer to the hidden layer, and constructs the hidden layer to the output layer in the form of weighted summation, and the output is the actuator and evaluator, that is, the adaptive control parameters and value functions defined above. The input of the input layer is the augmented state vector Θ(t), which is passed to the hidden layer, and then the hidden layer uses Θ(t) to calculate the output information of the layer, and the weight function of the input layer to the hidden layer is a radial basis function, that is, $\Phi \left(t\right)=\left[\phi_{1}\left(t\right),\cdots ,\phi_{h}\left(t\right)\right]$, and


$$\Phi_{j}\left(t\right)=\exp\left(-\frac{{\left\|\Theta \left(t\right)-\mu_{j}\left(t\right)\right\|}^{2}}{2\sigma_{j}^{2}\left(t\right)}\right),\;j=1,2,3,\ldots ,h$$ (30)

where: $\mu_{j}$ and$\sigma_{j}$ are the center and width of the radial basis function, respectively, and the center vector is defined as follows


$$\mu_{j}\left(t\right)={\left[\mu_{1j},\mu_{2j},\mu_{3j}\right]}^{T}$$ (31)

The third layer is the output layer including Actor and Critic, which is constructed in the form of a simple and direct weighted summation, and the adaptive PID controller parameters of the output can be expressed as


$$\begin{array}{c} K_{P}\left(t\right)=\sum_{j=1}^{h}w_{j}^{P}\left(t\right)\Phi_{j}\left(t\right)\\ K_{I}\left(t\right)=\sum_{j=1}^{h}w_{j}^{I}\left(t\right)\Phi_{j}\left(t\right)\\ K_{D}\left(t\right)=\sum_{j=1}^{h}w_{j}^{D}\left(t\right)\Phi_{j}\left(t\right) \end{array}$$ (32)

where: $w_{j}^{P}\left(t\right)$, $w_{j}^{I}\left(t\right)$ and $w_{j}^{D}\left(t\right)$ are the weighting coefficients between the *j-th* hidden layer node and the corresponding output Actor. The value function of Critic can be expressed as:


$$V\left(t\right)=\sum_{j=1}^{h}v_{j}\left(t\right)\Phi_{j}\left(t\right)$$ (33)

where: $v_{j}\left(t\right)$ is the weighting coefficient between the *j-th* hidden layer node and the output layer Critic.

The weight matrices from the input layer to the hidden layer and the hidden layer to the output layer can be calculated by the learning algorithm based on gradient descent. First, the reward signal r(·) in this paper is defined as:


$$r\left(x\left(t\right),u\left(t\right)\right)=\frac{1}{2}{\left(y_{d}\left(t+1\right)-y\left(t+1\right)\right)}^{2}$$ (34)

Then according to Eq 29, the timing differential error $\delta_{TD}\left(t\right)$ can be expressed as:


$$\delta_{TD}\left(t\right)=\frac{1}{2}{\left(y_{d}\left(t+1\right)-y\left(t+1\right)\right)}^{2}+\gamma V\left(t+1\right)-V\left(t\right)$$ (35)

According to the preceding definition, the cost function in this paper can be expressed as:


$$J\left(t\right)=\frac{1}{2}\delta_{TD}^{2}\left(t\right)$$ (36)

Therefore, the partial differential equation of the cost function with respect to the individual output weight matrices can be described as follows


$$\begin{array}{c} w_{j}^{P}\left(t+1\right)=w_{j}^{P}\left(t\right)-\alpha_{w}^{P}\frac{\partial J\left(t\right)}{\partial w_{j}^{P}\left(t\right)}\\ {}[12pt]w_{j}^{I}\left(t+1\right)=w_{j}^{I}\left(t\right)-\alpha_{w}^{I}\frac{\partial J\left(t\right)}{\partial w_{j}^{I}\left(t\right)}\\ {}[12pt]w_{j}^{D}\left(t+1\right)=w_{j}^{D}\left(t\right)-\alpha_{w}^{D}\frac{\partial J\left(t\right)}{\partial w_{j}^{D}\left(t\right)} \end{array}$$ (37)

where: $\alpha_{w}^{P}$, $\alpha_{w}^{I}$ and $\alpha_{w}^{D}$ are learning rates, and according to the defined cost function of Eq 36, the partial derivative in Eq 37 can be obtained by finding the partial derivative one by one, and the solution process is expressed as:


$$\begin{array}{c} \frac{\partial J\left(t\right)}{\partial w_{j}^{P}\left(t\right)}=\frac{\partial J\left(t\right)}{\partial \delta_{TD}\left(t\right)}\frac{\partial \delta_{TD}\left(t\right)}{\partial y\left(t+1\right)}\frac{\partial y\left(t+1\right)}{\partial u\left(t\right)}\frac{\partial u\left(t\right)}{\partial K_{P}\left(t\right)}\frac{\partial K_{P}\left(t\right)}{\partial w_{j}^{P}\left(t\right)}\\ \;=\delta_{TD}\left(y\left(t\right)-y\left(t-1\right)\right)\Phi_{j}\left(t\right)\frac{\partial y\left(t+1\right)}{\partial u\left(t\right)} \end{array}$$ (38)

$$\begin{array}{c} \frac{\partial J\left(t\right)}{\partial w_{j}^{I}\left(t\right)}=\frac{\partial J\left(t\right)}{\partial \delta_{TD}\left(t\right)}\frac{\partial \delta_{TD}\left(t\right)}{\partial y\left(t+1\right)}\frac{\partial y\left(t+1\right)}{\partial u\left(t\right)}\frac{\partial u\left(t\right)}{\partial K_{I}\left(t\right)}\frac{\partial K_{I}\left(t\right)}{\partial w_{j}^{I}\left(t\right)}\\ \;=-\delta_{TD}e\left(t\right)\Phi_{j}\left(t\right)\frac{\partial y\left(t+1\right)}{\partial u\left(t\right)} \end{array}$$ (39)

$$\begin{array}{c} \frac{\partial J\left(t\right)}{\partial w_{j}^{D}\left(t\right)}=\frac{\partial J\left(t\right)}{\partial \delta_{TD}\left(t\right)}\frac{\partial \delta_{TD}\left(t\right)}{\partial y\left(t+1\right)}\frac{\partial y\left(t+1\right)}{\partial u\left(t\right)}\frac{\partial u\left(t\right)}{\partial K_{D}\left(t\right)}\frac{\partial K_{D}\left(t\right)}{\partial w_{j}^{D}\left(t\right)}\\ \;=\delta_{TD}\left(y\left(t\right)-2y\left(t-1\right)+y\left(t-2\right)\right)\Phi_{j}\left(t\right)\frac{\partial y\left(t+1\right)}{\partial u\left(t\right)} \end{array}$$ (40)

From Eq 38 to Eq 40, it can be seen that the above partial derivatives all require prior knowledge of the Jacobian matrix of the known system, and according to assumptions 2, the sign of the Jacobian matrix is known, so this paper calculates the Jacobian matrix according to the equation established by the following identity.


ε=|ε|sign(ε) (41)

where: sign(·) is a symbolic function.


$$sign\left(\varepsilon \right)=\left\{ \;\;\;\begin{array}{c} \;1,\;\varepsilon >0\\ \;0,\;\varepsilon =0\\ -1,\;\varepsilon <0 \end{array}\right.$$ (42)

Then let $\frac{\partial y\left(t+1\right)}{\partial u\left(t\right)}$ be


$$\frac{\partial y\left(t+1\right)}{\partial u\left(t\right)}=\left|\frac{\partial y\left(t+1\right)}{\partial u\left(t\right)}\right|sign\left(\frac{\partial y\left(t+1\right)}{\partial u\left(t\right)}\right)$$ (43)

Since $sign\left(\frac{\partial y\left(t+1\right)}{\partial u\left(t\right)}\right)$ is known, for $\left|\frac{\partial y\left(t+1\right)}{\partial u\left(t\right)}\right|$, it can

be included in the learning rates such as $\alpha_{w}^{P}$, $\alpha_{w}^{I}$ and $\alpha_{w}^{D}$ [28]. Similarly, the radial basis function center and width of the hidden layer of a neural network can be updated online by the following adaptive law.


$$\mu_{ij}\left(t+1\right)=\mu_{ij}\left(t\right)-\alpha_{\mu }\frac{\partial J\left(t\right)}{\partial \mu_{ij}\left(t\right)}=\mu_{ij}\left(t\right)+\alpha_{\mu }\delta_{TD}\left(t\right)v_{j}\left(t\right)\Phi_{j}\left(t\right)\frac{\Theta_{i}\left(t\right)-\mu_{ij}\left(t\right)}{\sigma_{j}^{2}\left(t\right)}$$ (44)

$$\sigma_{j}\left(t+1\right)=\sigma_{j}\left(t\right)-\alpha_{\sigma }\frac{\partial J\left(t\right)}{\partial \sigma_{j}\left(t\right)}=\sigma_{j}\left(t\right)+\alpha_{\sigma }\delta_{TD}\left(t\right)v_{j}\left(t\right)\Phi_{j}\left(t\right)\frac{{\left\|\Theta_{i}\left(t\right)-\mu_{ij}\left(t\right)\right\|}^{2}}{\sigma_{j}^{3}\left(t\right)}$$ (45)

where: $\alpha_{\mu }$ and $\alpha_{\sigma }$ are the learning rate parameter.

In addition, the output weight matrix of Critic under the RBF neural network structure can be updated online by the following adaptive law.


$$v_{j}\left(t+1\right)=v_{j}\left(t\right)-\alpha_{v}\frac{\partial J\left(t\right)}{\partial v_{j}\left(t\right)}=v_{j}\left(t\right)+\alpha_{v}\delta_{TD}\left(t\right)\Phi_{j}\left(t\right)$$ (46)

where: $\alpha_{v}$ is the learning rate parameter that outputs the weight.

The design steps of reinforcement learning adaptive PID controller based on the Actor-Critic framework are shown in Table 1. The implementation process of Algorithm 1 requires setting some essential control parameters.

**Table 1:** Reinforcement learning adaptive PID controller design steps.

Algorithm 1. Design steps of reinforcement learning adaptive PID controller based on Actor-Critic framework
1. t=0, Initialize control input signal u(0) and reference input signal $y_{d}\left(0\right)$
2. Initialize the control parameters $w_{j}^{P}$, $w_{j}^{I}$, $w_{j}^{D}$, $v_{j}\left(0\right)$, $\mu_{ij}\left(0\right)$ and $\sigma_{j}\left(0\right)$, set the learning rates $\alpha_{w}$,$\alpha_{v}$,$\alpha_{\mu }$ and$\alpha_{\sigma }$
3. **for** t = 1:EndTime
4. The system output y(t) is measured and the output error is calculated according to $e\left(t\right)=y_{d}\left(t\right)-y\left(t\right)$
5. Calculation of kernel radial basis function of the hidden layer of RBF neural network structure (equation (30))
6. Calculate the output of the Actor at *t* moment by equation (32) to obtain the PID controller parameters, and calculate the output value function V(t) of the Critic at *t* moment by equation (33).
7. Obtain the control increment Δu(t) at the current moment by equation (33):
8. The control signal u(t)=u(t−1)+Δu(t) at the current time is calculated by equation (32), and it is input to the controlled puncture system, while the system output y(t+1) at the next time is generated
9. Based on the system output, build the next instantaneous expansion state: $\theta \left(t+1\right)={\left[e\left(t+1\right),-\Delta y\left(t+1\right),-\Delta^{2}y\left(t+1\right)\right]}^{T}$
10. Calculate the output value function V(t+1) of Critic at the time t + 1 according to equation (33)
11. Calculate the timing differential error $\delta_{TD}\left(t\right)$ according to equation (35)
12. Update the weight coefficients of the value function according to equation (37), (39), and (40) and the weight coefficients of the new PID parameter according to equation (45)
13. Update the center and width values of the RBF kernel function according to equation (44) and equation (45).
14. end for
15. End of Algorithm 1.

**Fig 10:**
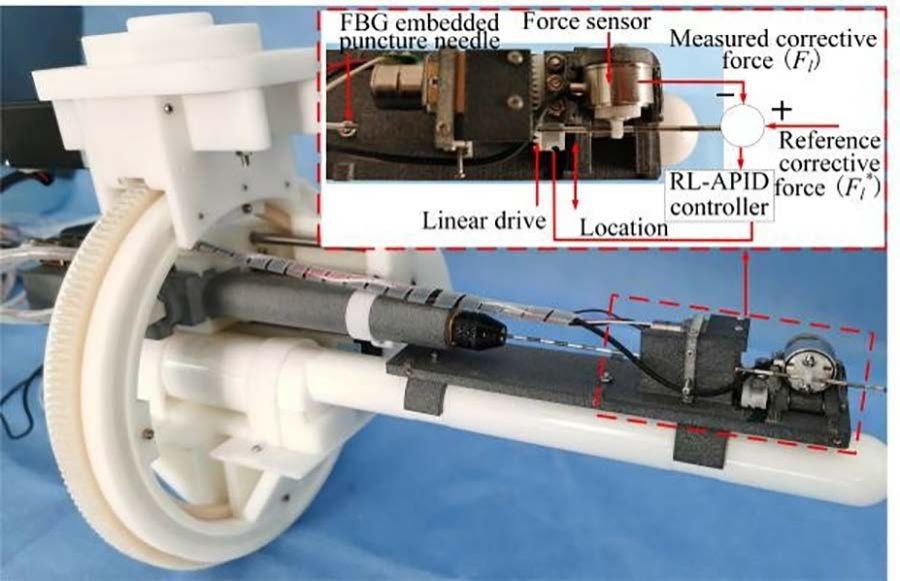
End effector closed-loop control system structure.

In this paper, given parameters *K*,*F*_*cutting*_ and measuring tip trajectory $u_{t}(d)$, the corrective force *F*_l_* magnitude of the reference can be calculated according to Eq 19. Therefore, the reference corrective force is used as the input of the reinforcement learning adaptive PID controller, the correction force measured by the actual system is used as feedback, the controller output is converted into the correction force through the linear drive device, and the closed-loop control system structure of the end effector is shown in Fig 10.

**Fig 11:**
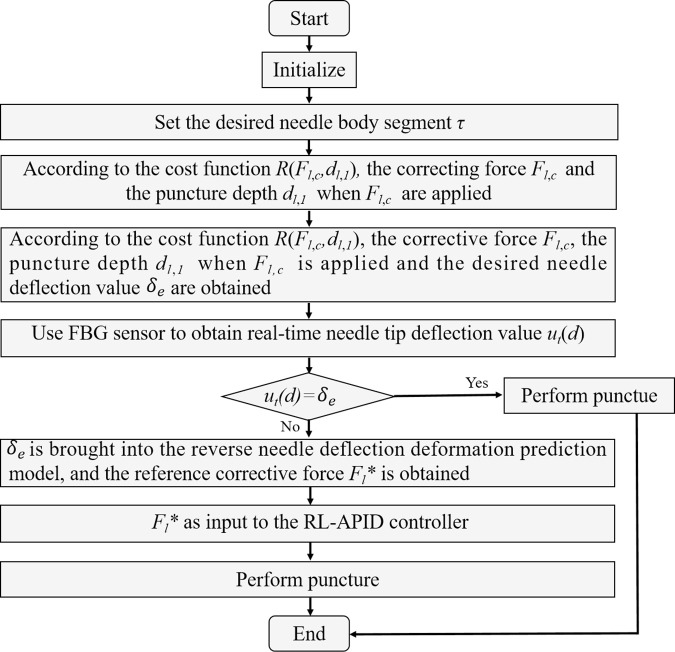
Puncture control strategy of prostate BT.

In summary, the puncture control strategy of the transrectal prostate BT robot is shown in Fig 11.

## Results and discussion

**Fig 12:**
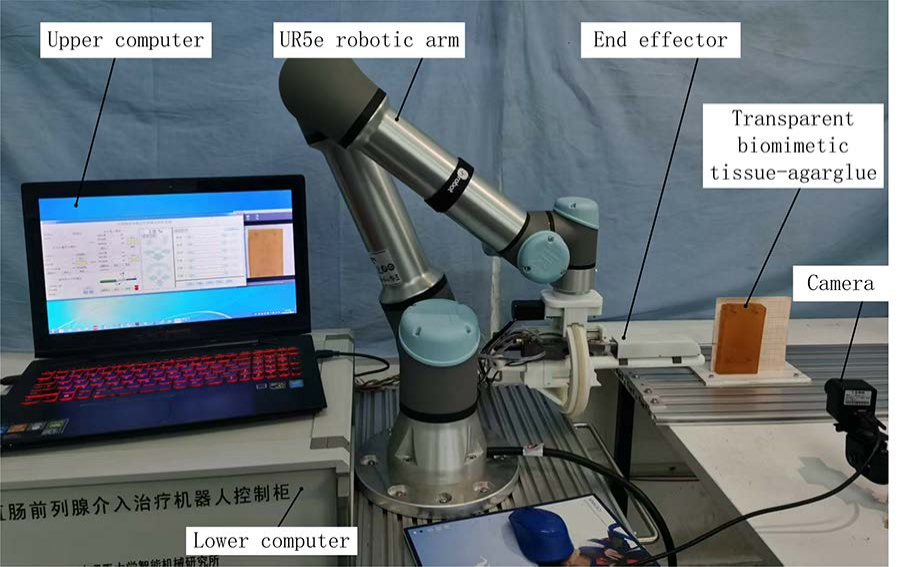
Control system block diagram.

In this chapter, the closed-loop control system experiment and comparative analysis study will be carried out to evaluate the feasibility and robustness of the proposed control method. In this paper, a robotic puncture platform is set up, as shown in Fig 12.

**Fig 13:**
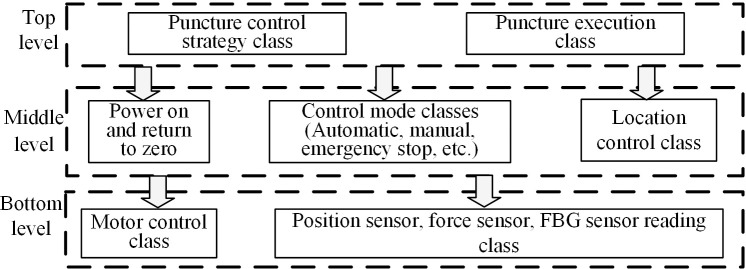
Control system block diagram.

The whole system consists of UR5e manipulator, end effector, upper computer and lower computer. The UR5e robotic arm is mainly used for the initial positioning of the actuator end. The structure of the robot control system is shown in Fig 13.

**Fig 14:**
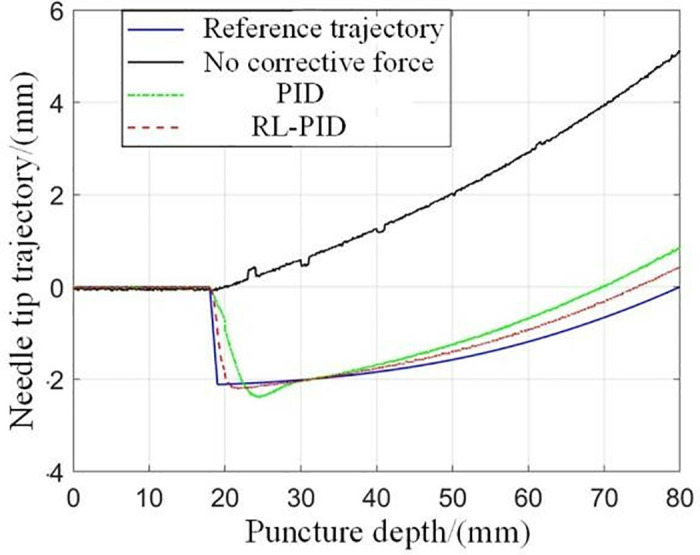
Comparison of puncture experiments results.

**Fig 15:**
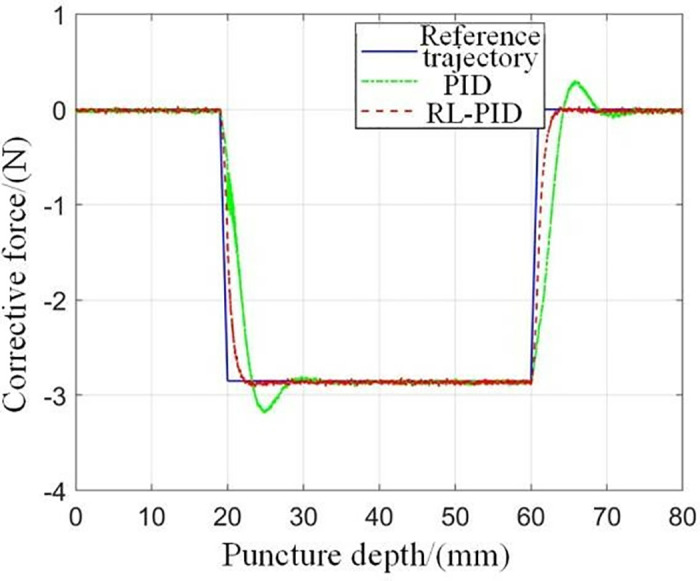
Comparison between the predicted correction force of the model and the actually applied correction force.

**Fig 16:**
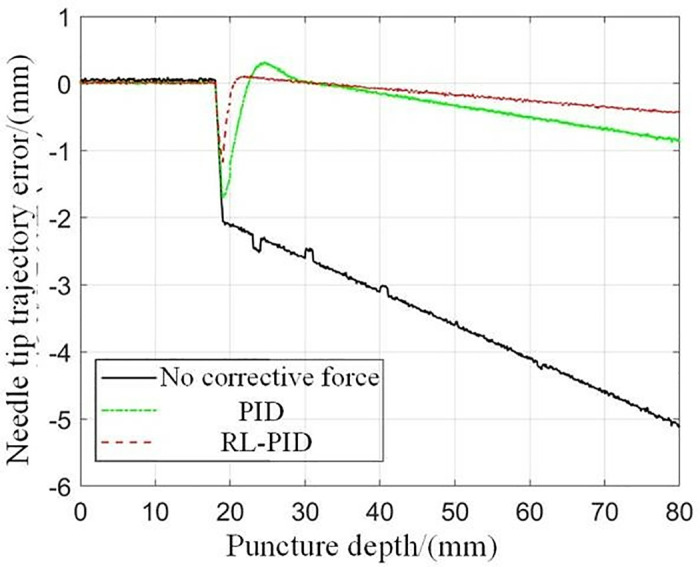
Needle tip trajectory error.

When the puncture objects are the same, the results of the experiments using different puncture strategies are compared and analyzed, and the traditional PID control method and the adaptive PID control method based on reinforcement learning proposed in this paper are used to carry out the puncture experiment, and the puncture depth of each puncture is 80 mm. The first group: without corrective force, the rectum and beef tissue were punctured at a depth of 80 mm; The second group: puncture rectum and beef tissue with traditional PID control method, the puncture depth is 80 mm, and the initial PID control parameters are set as follows: $K\left(0\right)={\left[0,0,0\right]}^{T}$; The third group: the adaptive PID control method of reinforcement learning was used to puncture rectal and beef tissues with a puncture depth of 80 mm, and the main parameters of RL-APID were selected as follows: $\alpha_{w}=0.13,\alpha_{v}=0.35,\alpha_{\mu }=0.027,\alpha_{\sigma }=0.015,\gamma =0.90$ Each group of experiments was repeated 5 times, and the average value was taken as the final result, as shown in Figs 14 and 15. From Fig 16, it can be seen that the needle tip trajectory will gradually deviate from the reference trajectory when no corrective force is applied, and the deviation will be significantly reduced after the corrective force is applied.

**Fig 17:**
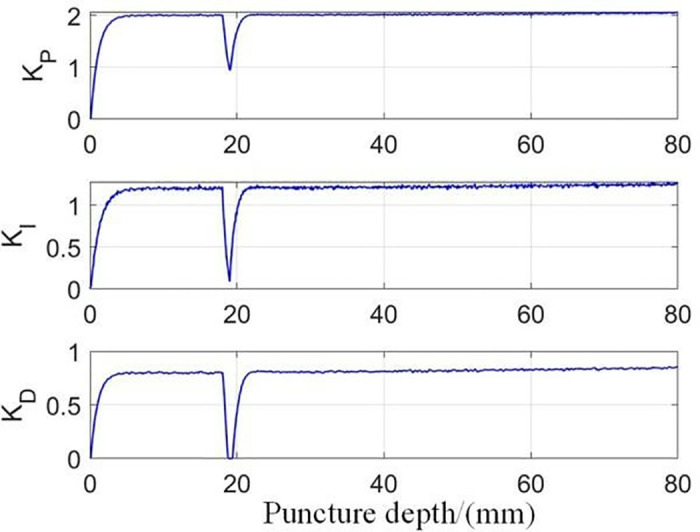
Adaptive PID parameter variation.

In addition, it can be seen that the RL-APID tracking error is smaller, the dynamic control performance is more stable when the reference trajectory jumps, there is no excessive overshoot or jitter, and the traditional PID will produce a relatively large overshoot and jitter during the trajectory jump, which is not conducive to the smooth progress of the puncture, in addition, the trajectory tracking error Fig 16 shows that RL-APID also has higher trajectory tracking accuracy, and the lateral driving force of the RL-APID control output can significantly reduce the deviation of the needle puncture process. **Fig 17** shows the process of adaptive adjustment of RL-APID parameters during the puncture process. From Fig 16, it can be seen that piercing by adaptive PID control method of reinforcement learning can reduce the needle deflection value by 90% at a puncture depth of 80 mm, and has higher puncture accuracy.

**Fig 18:**
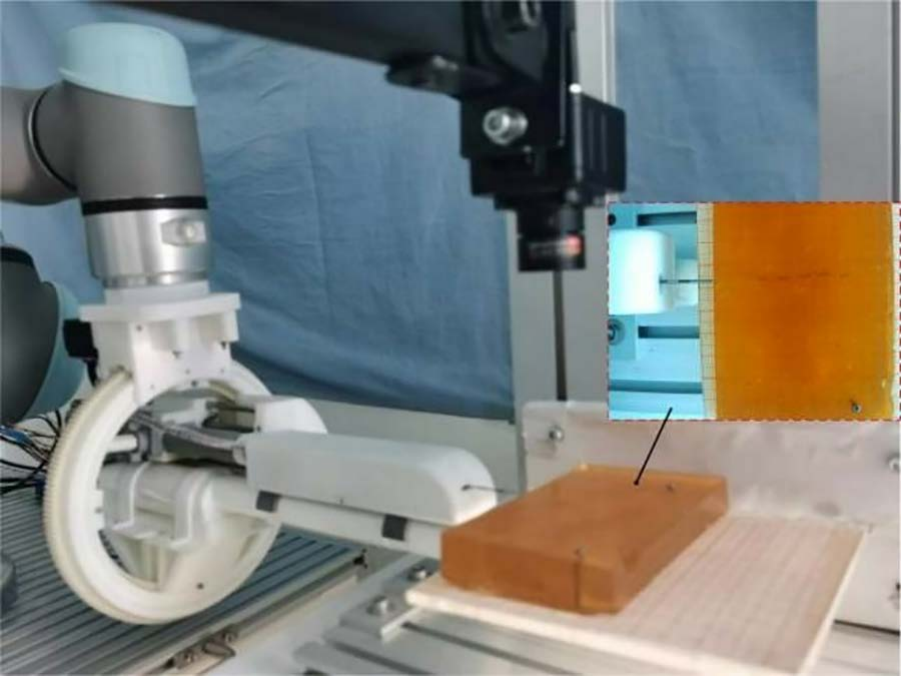
Biomimetic tissue seed implantation experiment.

Since the material of the seeds in the real tissue could not be seen, the transparent biomimetic tissue-agar glue was used for the seed implantation experiment, as shown in Fig 18, the relative coordinate values of the seeds implantation points were obtained by image processing of the seeds implantation points by MATLAB, and 5 seeds were implanted each time, and the experiment was repeated 5 times to take the average of its data. By comparing the theoretical coordinate values of particles with the actual coordinate values, the deviation value between the two is obtained, as shown in Table 2. Finally, the absolute error of average seeds implantation is 1.96 mm and the standard error is 0.56 mm, and the seeds implantation accuracy meets the clinical requirements of 3–6 mm [4].

**Table 2:** Seed implantation experiment results.

Scheme	Theoretical coordinate value	Actual coordinate value	Deviation value
1	(3.0, 16.0)	(3.8, 15.7)	1.2
2	(3.0, 32.0)	(4.2, 33.2)	1.5
3	(3.0, 48.0)	(4.6, 47.8)	1.8
4	(3.0, 64.0)	(5.4, 65.2)	2.6
5	(3.0, 80.0)	(5.6, 78.2)	2.8

## Conclusions

In this paper, a corrective force-based puncture control strategy is proposed that uses only the corrective force drive to minimize the deflection value of the needle at the final puncture depth.The puncture control strategy is divided into two stages: the preoperative needle trajectory planning stage and the intraoperative puncture strategy adjustment stage. In the preoperative needle trajectory planning stage, the optimal needle tip trajectory and puncture parameters were obtained based on the needle deflection prediction model. In the stage of adjusting the intraoperative puncture strategy, a reverse needle tip deflection prediction model was constructed, and the value of the corrective force was compensated intraoperatively, and the traditional PID control and the adaptive PID control method based on reinforcement learning were used to control the application of the correction force to achieve accurate puncture. In addition, the effectiveness of the puncture control strategy is verified and compared based on the experimental platform o0066 prostate BT robot, and the puncture experimental results show that the adaptive PID control method based on reinforcement learning can effectively reduce the deflection value of the needle tip, and has smaller overshoot and jitter than the traditional PID control method, and has higher puncture accuracy. In the seeds implantation experiment, the average implantation error of seeds implantation is 1.96 mm and the standard error is 0.56 mm, which can meet the clinical and design index requirements.

## Supporting information

S1 File https://doi.org/10.6084/m9.figshare.28300652 (RAR).
(DOCX)
