# Supplementary material for: Research on prostate brachytherapy puncture control strategy based on adaptive PID control with FBG sensors
Source: PLoS One. 2025 Aug 13;20(8):e0329065. doi: 10.1371/journal.pone.0329065 (PMC12349729; doi:10.1371/journal.pone.0329065)
Supplement: S1 File — https://doi.org/10.6084/m9.figshare.28300652 (RAR). (DOCX) [file pone.0329065.s001.docx]

**Supporting information**

S1 File：[https://doi.org/10.6084/m9.figshare.2](https://doi.org/10.6084/m9.figshare.28300652)[8300652](https://doi.org/10.6084/m9.figshare.28300652) (RAR)
